# Reply to Erren et al. and Martin-Olalla and Mira: What constitutes circadian burden? An empirically grounded approach to long-term summary metrics

**DOI:** 10.1073/pnas.2533444123

**Published:** 2026-04-17

**Authors:** Lara Weed, Jamie M. Zeitzer

**Affiliations:** ^a^Department of Bioengineering, Stanford University, Stanford, CA 94305; ^b^Department of Psychiatry and Behavioral Sciences, Stanford University, Stanford, CA 94305

Models of the human circadian clock use ocular light exposure patterns to predict circadian timing (phase). In our recent work on the circadian health impacts of time policy (1), we simulated county-level light exposure patterns across the continental United States and entered them into models predicting circadian timing under permanent Standard Time, Daylight Saving Time, and biannual switching. Since these models produce daily phase estimates, yet population health outcomes are annual, we required a summary circadian measure to bridge temporal scales. Among many theoretical approaches, we intentionally selected a simple and physiologically grounded metric.

To remain synchronized to the 24-hour day, the circadian clock, which has an intrinsic period of ~24.2 h (2), is adjusted by light, being advanced by morning light and delayed by evening light (3). Thus, every day, there is a push–pull on the circadian clock, with the magnitude determined by intrinsic clock parameters (e.g., period length, light sensitivity) and light exposure features (e.g., intensity, timing, wavelength composition). Our cumulative absolute circadian shifting metric quantitates compensatory regulation magnitude without assigning directionality, and functions analogously to displacement measures summarizing the degree of alignment effort imposed across the year.

Two recent letters have raised questions about the interpretation and methodology of our study (4, 5). While we appreciate their interest in our work, Martin-Olalla and Mira’s (4) commentary appears to stem from a misinterpretation of both the sampling structure of our example code and the intended meaning of the shifting metric. They mistakenly interpret the code’s discrete sampling resolution as noise rather than as a product of a fixed sampling grid. They then attempt to examine seasonal trends in *signed* daily shifting at a single location but do not recognize that shifting is regulatory and should sum to zero in an entrained system. This signed metric is also not what we used in our health models. While these misinterpretations lead them to incorrect conclusions about model behavior, their commentary nevertheless raises an important broader question: how should we quantify circadian impact over extended periods, and under what conditions does that impact constitute “burden”?

The magnitude of any “impact” or “burden” ultimately depends on an outcome of interest. In our work, we evaluated population-level health outcomes to tangibly quantify the impact of circadian phase shifting exposure. Although the mechanistic links between chronic circadian disruption and poor health are not yet fully understood, several theoretical constructs, including weakened coupling among downstream oscillators and misalignment between internal and external time, have been posited to explain the relationship. We emphasize that this does not establish causation, nor does it imply that phase adjustments themselves are intrinsically harmful. Instead, our metric operationalizes misalignment exposure in a manner consistent with empirical circadian science, enabling statistical examination of its associations with real-world outcomes. Alternate metrics, such as those based in model latent states, typically require stronger assumptions or lack direct physiological grounding. We therefore adopted the most parsimonious and experimentally-anchored approach available given current data and modeling constraints.

More broadly, Erren et al. raised concerns regarding the interpretation of ecological analyses linking modeled circadian measures with county-level health data (5). The impact of time policy on human health is complex (6) and nigh impossible to answer with the traditional case–control study. When Daylight Saving Time was introduced, the biological impact of light exposure and behavioral timings were poorly understood. In the past several decades, however, research in this area has expanded substantially. Most of the extant literature on time policy focuses on the fall and spring transition points in countries that have biannual shifts (7), though this does not address what could occur during yearlong exposure to specific time policies, just what occurs at the transition points. Comparing countries with different time policies but being located at the same latitude is also a highly problematic approach given genetic, cultural, and weather variations that can have interactive effects on behavior and health. To avoid these pitfalls, we compared model-estimated health impacts of transitioning to year-round daylight saving time or year-round standard time, as compared to our current U.S. policy of biannual shifting (1). To do so, we examined county-level data and modeled the impact of idealized light exposure patterns on circadian rhythms; both approaches make many assumptions about health and behavior.

Importantly, county-level prevalence does not directly translate to individual outcomes. For any given individual, specific self-selected light exposure patterns will have significant differential impacts on their circadian system (8), and this does not even consider individual vulnerabilities and preexisting conditions that would change the likelihood of specific diseases. Our work also does not examine the effect of time policy on behaviors that would be likely to directly (e.g., changes in exercise patterns) or indirectly (e.g., changes in employment opportunities in economically vulnerable populations) impact human health. It will be crucial for future research to integrate these impacts on health.

Understanding how time policy influences human biology remains an important and unresolved scientific question. Our analyses provide one approach for examining this question by integrating physiologically grounded circadian models with population-scale health data. While such analyses necessarily involve assumptions and should not be interpreted as establishing individual-level causation, they allow large-scale hypotheses about the interaction between time policy, circadian regulation, and health to be quantitatively explored. We hope this work contributes to an ongoing scientific dialog about the biological consequences of societal timing systems and helps motivate future interdisciplinary research on this topic.
